# Salutatory

**Published:** 1854-01

**Authors:** 


AFTER a temporary suspension of our Journal, occasioned by the destruction of the office by fire at which it was published, we have the pleasure at length of presenting to our readers the first number of a new series, in a style every way improved. The two numbers still due, for November and December, will be issued with the least practicable delay, and we have the promise of the Publisher, that the work shall hereafter appear punctually at the beginning of the month. It is with a feeling of great satisfaction that we announce that we have secured a corps of contributors, whose experience and readiness as practitioners and writers insure us an ample supply of original matter. Our colleagues in the University of Louisville are pledged to the support of the Journal, and in addition to these we have many friends in the profession who have promised us contributions.

The *Western Journal* is now the oldest journal of medicine published in the Valley of the Mississippi, and, regarded as a continuation of Dr. Drake's Journal, which was merged in it, the oldest in the United States. We believe, of all our brethren of the medical press in our country, we were earliest to occupy the editorial chair. When our present enterprise was set on foot fourteen years ago, there was no medical journal in progress in all the States west of the mountains, and it seemed to be loudly called for by the profession. Since that time the number projected has been equal to one a year; but notwithstanding this unprecedented multiplication, we believe the friends of our Journal would not willingly see it suspended. It is with this persuasion, and in the hope of being able to serve and advance our profession, that we continue in a post from which twenty years of laborious service would seem to entitle us to an honorable discharge.

As the result of a good deal of experience and inquiry, we have determined to reduce somewhat the size, and materially the price of our Journal. Heretofore, for twelve numbers of 92 pages, the subscription has been $5 per annum; it is now only $3 for twelve numbers of 80 pages.

This number is sent to many physicians who are not subscribers. If they are satisfied with the work, they will please forward their names to the Publisher, who alone is interested in its pecuniary concerns.

